# Simulation of Greenhouse Climate Monitoring and Control with Wireless Sensor Network and Event-Based Control

**DOI:** 10.3390/s90100232

**Published:** 2009-01-08

**Authors:** Andrzej Pawlowski, Jose Luis Guzman, Francisco Rodríguez, Manuel Berenguel, José Sánchez, Sebastián Dormido

**Affiliations:** 1 Department of Languages and Computation, University of Almería; Ctra. Sacramento s/n, 04120, Almería, Spain; E-Mails: ap245@ual.es; frrodrig@ual.es; beren@ual.es; 2 Department of Computer Science and Automatic Control, UNED C/. Juan del Rosal, 16, 28040, Madrid, Spain; E-Mails: jsanchez@dia.uned.es; sdormido@dia.uned.es

**Keywords:** Event-based control, Wireless Sensor Network, greenhouse climate control

## Abstract

Monitoring and control of the greenhouse environment play a decisive role in greenhouse production processes. Assurance of optimal climate conditions has a direct influence on crop growth performance, but it usually increases the required equipment cost. Traditionally, greenhouse installations have required a great effort to connect and distribute all the sensors and data acquisition systems. These installations need many data and power wires to be distributed along the greenhouses, making the system complex and expensive. For this reason, and others such as unavailability of distributed actuators, only individual sensors are usually located in a fixed point that is selected as representative of the overall greenhouse dynamics. On the other hand, the actuation system in greenhouses is usually composed by mechanical devices controlled by relays, being desirable to reduce the number of commutations of the control signals from security and economical point of views. Therefore, and in order to face these drawbacks, this paper describes how the greenhouse climate control can be represented as an event-based system in combination with wireless sensor networks, where low-frequency dynamics variables have to be controlled and control actions are mainly calculated against events produced by external disturbances. The proposed control system allows saving costs related with wear minimization and prolonging the actuator life, but keeping promising performance results. Analysis and conclusions are given by means of simulation results.

## 1. Introduction

Nowadays, the agro-alimentary sector is incorporating new technologies due to the large production demands and the diversity, quality, and market presentation requirements. A technological renovation of the sector is being required where the control engineering plays a decisive role. Automatic control and robotics techniques are incorporated in all the agricultural production levels: planting, production, harvest, post-harvest processes, and transportation. Modern agriculture is subjected to regulations in terms of quality and environmental impact, and thus it is a field where the application of automatic control techniques has increased substantially during the last years [1-5].

As it is well-known, greenhouses have a very extensive surface where the climate conditions can vary at different points (spatial distributed nature). Despite of that feature, it is very common to install only one sensor for each climatic variable in a fixed point of the greenhouse as representative of the main dynamics of the system. One of the reasons is that typical greenhouse installations require a large amount of wire to distribute sensors and actuators. Therefore, the system becomes complex and expensive and the addition of new sensors or actuators at different points in the greenhouses is thus quite limited.

In the last years, Wireless Sensor Networks (WSN) are becoming an important solution to this problem [6-7]. WSN is a collection of sensor and actuators nodes linked by a wireless medium to perform distributed sensing and acting tasks [8]. The sensor nodes collect data and communicate over a network environment to a computer system, which is called, a base station. Based on the information collected, the base station takes decisions and then the actuator nodes perform appropriate actions upon the environment. This process allows users to sense and control the environment from anywhere [7]. There are many situations in which the application of the WSN is preferred, for instance, environment monitoring, product quality monitoring, and others where supervision of big areas is necessary [9]. In this work, WSN are used in combination with event-based systems to control the inside greenhouse climate.

On the other hand, event-based systems are becoming increasingly commonplace, particularly for distributed real-time sensing and control. A characteristic application running on an event-based operating system is that where state variables will typically be updated asynchronously in time, for instance, when an event of interest is detected or because of delay in computation and/or communication [10]. Event-based control systems are currently being presented as solutions to many control problems [10-13]. In event-based control systems, the proper dynamic evolution of the system variables is what decides when the next control action will be executed, whereas in a time-based control system, the autonomous progression of the time is what triggers the execution of control actions. The fundamental reason for the predominance of the time-based control systems has been based on the existence of a well established theory for control systems with a constant sampling time [14]. However, current distributed control systems also impose restrictions on the architecture of the system that makes difficult the adoption of a paradigm based on events activated per time. For instance, in the case of closed-loop control using computer networks or buses (such as field bus, local network area, or Internet), where asynchronous communication is required. An alternative to these approaches consists of using event-based controllers that are not restricted to the synchronous occurrence of controller actions. The employment of synchronous sampling period is one of the severest conditions that control engineers follow for implementation tasks. Many examples can be found, such as mobile phones, printing devices, or PDA's. The complexity of these devices (processes), as well as the complexity of the controller, is increasing very fast. These requirements can be reduced with event-based controllers, where the control actions can be executed in an asynchronous way [15].

Control problems in greenhouses are mainly focused on fertirrigation and climate systems. The fertirrigation control problem is usually solved providing the amount of water and fertilizers required by the crop. The climate control problem consists in keeping the greenhouse temperature and humidity in specific ranges despite of disturbances. Adaptive and feedforward controllers are commonly used for the climate control problem. Therefore, fertirrigation and climate systems can be represented as event-based control problems where control actions will be calculated and performed when required by the system, for instance, when water is required by the crop or when ventilation must be closed due to changes in outside weather conditions. Furthermore, such as discussed above, with event-based control systems a new control signal is only generated when a change is detected in the system. That is, the control signal commutations are produced only when events occur. This fact is very important for the actuator life and from an economical point of view (reducing the use of electricity or fuel), especially in greenhouses where commonly actuators are composed by mechanical devices controlled by relays.

Therefore, this paper presents the combination of WSN and event-based control systems to be applied in greenhouses. The main focus of this paper is therefore the presentation of a complex real application where using a WSN, as an emerging technology and Event-Based Control, as a new paradigm in process control, the following issues have been addressed:

- the issues posed to a multivariable, interacting control system by possibly faulty communications (as in a wireless context),
- the location of sensors to correctly represent, for the purpose of control, spatially distributed quantities,
- the efficient use of actuators, the term “efficient” referring also to correct use and wear minimization,
- the effects of event-based sampling.

As a first approximation, event-based control has been applied for temperature and humidity control issues. The main advantages of the proposed control problem in comparison with previous works is that promising performance results are reached reducing the use of wire and the changes on the control signals, what it is translated into reductions on costs and a longer actuator life. The ideas presented in this paper could be easily extrapolated, for instance, to building automation.

The work is organized as follows. Section 2 is devoted to describe the greenhouse climate control problem. Afterwards, the event-based system and WSN for the greenhouse temperature control is discussed. Simulations results are presented in section 4. Finally, some conclusions are given in Section 5.

## 2. Greenhouse Climatic Control Problem

### 2.1. Description of the Climatic Control Problem

Crop growth is mainly influenced by the surrounding environmental climatic variables and by the amount of water and fertilizers supplied by irrigation. This is the main reason why a greenhouse is ideal for cultivation, since it constitutes a closed environment in which climatic and fertirrigation variables can be controlled to allow an optimal growth and development of the crop. The climate and the fertirrigation are two independent systems with different control problems. Empirically, the requirements of water and nutrients of different crop species are known and, in fact, the first automated systems were those that controlled these variables. As the problem of greenhouse crop production is a complex issue, an extended simplification consists of supposing that plants receive the amount of water and fertilizers that they require at every moment. In this way, the problem is reduced to the control of crop growth as a function of climate environmental conditions [16, 17].

The dynamic behavior of the greenhouse microclimate is a combination of physical processes involving energy transfer (radiation and heat) and mass balance (water vapour fluxes and CO_2_ concentration). These processes depend on the outlet environmental conditions, structure of the greenhouse, type and state of the crop, and on the effect of the control actuators [18]. The main ways of controlling the greenhouse climate are by using ventilation and heating to modify inside temperature and humidity conditions, shading and artificial light to change internal radiation, CO_2_ injection to influence photosynthesis, and fogging/misting for humidity enrichment. A deeper study about the features of the climatic control problem can be found in [16].

The approach presented in this paper is applied to the climatic conditions of mild winter in Southern Europe (the data used for the simulations performed in this paper have been collected in a greenhouse from Southeastern Spain), where the production in greenhouses is made without CO_2_ enrichment and the demand of quality products is increasing every day. Considering the greenhouse structures, the commonest actuators, the crop types, and the commercial conditions of this geographical area, the main climate variables to control are the temperature and the humidity. The PAR (Photosynthetically Active Radiation: it is the spectral range from 400 to 700 Wm^2^, which is used by the plants as energy source in the photosynthesis process.) radiation is controlled with shade screens but its use is not much extended. So, this paper is focused on the temperature and humidity control problems.

### 2.2. Air Temperature Control

Plants grow under the influence of the PAR radiation (diurnal conditions), performing the photosynthesis process. Furthermore, temperature influences the speed of sugar production by photosynthesis, and thus radiation and temperature have to be in balance in the way that a higher radiation level corresponds to a higher temperature. Hence, under diurnal conditions, it is necessary to maintain the temperature in a high level, being optimal for the photosynthesis process. In nocturnal conditions, plants are not active (the crop does not grow); therefore it is not necessary to maintain such a high temperature. For this reason, two temperature set-points are usually considered: diurnal and nocturnal [19].

Due to the favorable climate conditions of Southeastern Spain, during the daytime the energy required to reach the optimal temperature is provided by the sun. In fact, the usual diurnal temperature control problem is the refrigeration of the greenhouse (with temperatures higher than the diurnal set-point) using natural ventilation to reach the optimal diurnal temperature. On the other side, the nocturnal temperature control problem is the heating of the greenhouse (with temperatures lower than the nocturnal set-point) using heating systems to reach the nocturnal optimal temperature. In Southeastern Spain, forced-air heaters are commonly used as heating systems.

In this work, the diurnal and nocturnal temperature control is analyzed to test the proposed event-based control. Therefore, typical temperature control systems with ventilation and heating are described in the following section.

**Figure 2.**
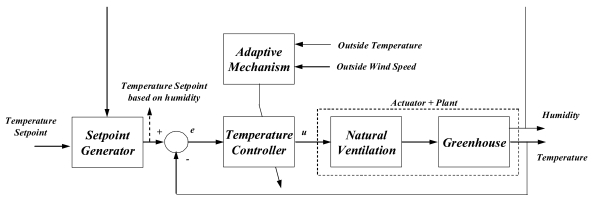
Diurnal gain scheduling controller with temperature setpoint generator based on humidity.

The natural ventilation determines the air exchange and air flow in the greenhouse as a consequence of the differences between outside and inside temperatures. The relationship between vents aperture and inside temperature is not linear [20], but instead of using a nonlinear control schema, it was decided to implement a gain-scheduling control algorithm based on linear models for each operating point (see Figure 2). Most commercial solutions include this kind of gain scheduling controllers to cope with both fast and slow changing dynamics due to disturbances. This controller consists in a gain-scheduling PI scheme where the controller values are changed based on some disturbances: outside temperature and wind speed. For the nocturnal temperature control, there exist numerous control strategies, but for this study an on/off control with dead/zone is used in forced-air heaters, which is the controller commonly used in conventional greenhouses. A full description of these algorithms can be found in [16].

### 2.3. Humidity Control

Water vapour inside the greenhouse is not one of the most important variables affecting the crop growth. However, the humidity control has a special interest, because high humidity may produce the appearance of diseases and decrease transpiration, and low humidity may cause hydric stress, closing the stomata, and thus reducing the photosynthesis due to a decrease in the CO_2_ assimilation. There are two problems involved in the humidity control: (1) the greenhouse inside temperature and the relative humidity are inversely related when the greenhouse air not mixed with the external air, generally colder and drier (when one of them increases the other one decreases and *vice versa*); (2) the same actuators are used for controlling temperature and humidity. The temperature control has the main priority because it affects to the crop growth directly. In order to keep the humidity within a determined range, the temperature set-point can be changed based on the inside relative humidity value. Hence, the humidity controller acts as a set-point generator being able to change the temperature set-point in small ranges. The modification of the temperature set-point value depends on actual humidity level, selecting a maximum allowable modification for a specific kind of crop. A lower temperature set-point allows evacuating the humid air as a consequence of the exchange with the outside air because it is drier than the internal air. However, a higher set-point provokes that the ventilation remains closed for longer periods of time keeping the water vapour of air inside the greenhouse, and so increasing the humidity [16-17].

## 3. WSN and Event-Based System for Greenhouse Climate Control

Before addressing the greenhouse climate control as an event-based control problem, some basic notions about level crossing sampling and event-based control are introduced.

### 3.1. Level Crossing Sampling and Event-Based Control

As discussed above, in an event-based control system the control actions are executed in an asynchronous way, that is, the sampling period is governed by system events and it is called event-based sampling. The event-based sampling is a very old idea [21] and indicates that the most appropriate method of sampling consists in transmitting information only when a significant change in the signal occurs justifying the acquisition of a new sample. During last years, researchers have demonstrated special interest on this sampling technique [22-25]. This kind of sampling method has received different names in the literature [15]: adaptive sampling, asynchronous delta-modulation, dead-band method, send-on-delta method, level crossing sampling, and Lebesgue sampling.

In spite of these names (in this paper the name *level crossing sampling* is selected), the basic principle is the same: the input signal is sampled when the absolute value of the difference between the current value, *x(t_s_)*, and the last sampled value of a signal, *x(t_k_)*, is bigger than a specific limit *δ*:

(1) $$\left|x\left(t_{k}\right)-x\left(t_{s}\right)\right|>\delta$$

**Figure 3.**
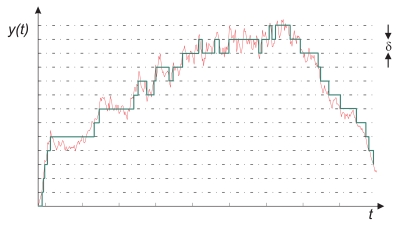
Level crossing method.

When the change in the signal is relatively small, the number of samples is significantly smaller than in a periodic sampling scheme, such as shown in Figure 3.

In an event-based system, the occurrence of an event, rather than the passing of the time, is what decides when a sample should be taken. The nature of the event could vary. Examples could be those as a measured signal crosses a certain limit or the arrival of a data packet to a node on a computer-based network [11].

When the sampling is event-triggered, event-based control systems can be considered as alternative for time-based control systems. Other names for these control systems are aperiodic or asynchronous control systems. In a general way, an event-based controller consists of two parts: an event detector and a controller. The event detector deals with indicating to the controller when a new control signal must be calculated due to the occurrence of a new event. For instance, the decision to calculate a new control signal could be if the absolute value of the difference between the current value of the error, *e(t_k_)*, and the value of the last error calculated, *e(t_s_)*, is greater than a limit *δ*, or when the time elapsed since the last sample, *h_act_*, exceeds a maximum limit *h_max_*:

(2) $$\left|e\left(t_{k}\right)-e\left(t_{s}\right)\right|>\delta \;\text{and}\;t_{k}-t_{s}\geq h_{\max}$$

From condition (2), the controller will be executed at the nominal sampling time *h_nom_* during transients (for instance, set point changes and load disturbances) or if the time difference between samples is bigger than the maximal sampling interval, *h_max_,* during steady state conditions [11]. The last condition, *t_k_* − *t_s_* ≥ *h_max_*, is a simple safety condition to ensure the control system stability due to sampling problems.

### 3.2. WSN and Event-Based Control in Greenhouses

As commented above, this paper is devoted to analyzing diurnal and nocturnal temperature control with natural ventilation and heating systems, and humidity control as a secondary control objective. Under diurnal conditions, the controlled variable is the inside temperature and the control signal is the vent opening. The use of natural ventilation produces an exchange between the inside and outside air, usually provoking a decrease in the inside temperature of the greenhouse. The controller must calculate the necessary vent opening to reach the desired set-point. As discussed in Section 2, the commonest controller used is a gain scheduling PI scheme where the controller parameters are changed based on some disturbances: outside temperature and wind speed. In the case of nocturnal temperature control, forced-air heaters are used to increase the inside temperature and an on/off control with dead/zone was selected as heating controller.

**Figure 4.**
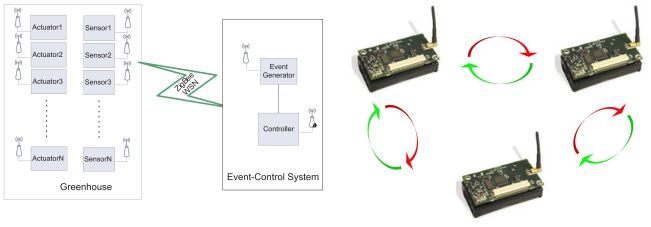
Event-Based Control and WSN

In this work, the idea is to combine WSN with event-based control such as shown in Figure 4. The sensors are distributed along the greenhouse including a level crossing sampling for each variable. That is, the greenhouse is provided with a WSN where each sensor will transmit data if the absolute value of the difference between the current value of the variable, *v(t_k_)*, and the previous value of the variable *v(t_s_)*, is greater than a limit *δ*. Therefore, the first step in this work was to calculate suitable limits δ for each greenhouse variable. Such as shown in next section, this limit has a direct influence on the event generation and on the quantity of transmitted data.

Table 1 shows the individual limits for the commonest variables used for control purposes. These limits of *δ* = 3% and *δ* = 5% were calculated based on the authors experience and after analyzing three years of data. The calculation of δ limit for each individual variable was performed studying its minimum and maximum values. The value of the change of each variable for *δ* = 3% and *δ* = 5% was determined calculating the 3% and 5% of the difference between the maximum and minimum values. Instead of choosing only one limit for each variable, these two different limits were evaluated to analyze their effects, such as presented in next section.

On the other hand, the control system is now composed by an event generator plus the original controllers. In this way, the gain scheduling and the on/off controllers will calculate a new control action only when an event is detected by the event generator. The typical event is that when the absolute value of the difference between the current value of the error, *e(t_k_)*, and the value of the error calculated previously, *e(t_s_)*, is greater than a limit *δ*. In accordance with equation (2), transmission is also made when the time elapsed since the last sample, *h_act_*, exceeds the limit *h_max_*. Furthermore, and such as commented above, the control depends on disturbances and other events will be also generated by changes of the corresponding disturbances. The events from the disturbances will be detected by the event generator according to the limits shown in Table 1. These events are analyzed in Section 4.

## 4. Simulation Results

### 4.1. Material and Methods

The simulations presented in this section have been performed using the greenhouse climatic model developed by [16] and the TrueTime MATLAB/Simulink toolbox [26], [27].

As commented above, the dynamic behavior of the microclimate inside the greenhouse is a combination of physical processes involving energy transfer (radiation and heat) and mass balance (water vapor fluxes and CO2 concentration). The greenhouse climate model developed in [16] capturates this dynamic behavior and it can be summarized as:

$$\frac{dX}{dt}=f(X,U,P,V,C,t),X(t_{i})=X_{i}$$

where *X* = *X(t)* is a *n_1_*-dimensional vector of greenhouse climate state variables (mainly the inside air temperature and humidity, CO_2_ concentration, PAR radiation, soil surface temperature, cover temperature, and plant temperature), *U* = *U(t)* is a *m*-dimensional vector of input variables (in this work natural vents and heating system), *D* = *D(t)* is an *o*-dimensional vector of disturbances (outside temperature and humidity, wind speed and direction, outside radiation, and rain), *V* = *V (t)* is a *p*-dimensional vector of system variables (related to transpiration, condensation, and other processes), *C* is a *q*-dimensional vector of system constants, *t* is the time, *X_i_* are the known states at the initial time *t_i_*, and *f* = *f(t)* is a nonlinear function based on mass and heat transfer balances.

The model has been developed using an object-oriented approach, so that the model hierarchy allows performing a modular parameter identification procedure. The values of the coefficients of the physical processes involved in the previous equation (which are the basis for the formulation of the nonlinear simulation models) have been obtained using both iterative search and genetic algorithms. A large set of input/output data has been used for calibration purposes covering different operating conditions obtained at the real greenhouse (data for six years).

The original data used for the model and for the simulations described in this paper were measured from a Parral greenhouse located at The Cajamar Foundation (El Ejido, Almería, South-East Spain). The covering material is a 200-micron thick PE film, laid on a galvanized steel structure. The air temperature thermoresistance sensors and the air relative humidity capacitive sensors were placed at the top of the crop. A meteorological station was installed outside at a height of 6 m for measurements of temperature, relative humidity, global and PAR radiation, rain, and wind speed and direction.

As commented above, the greenhouse climate model has been used in combination with the TrueTime toolbox. TrueTime is a tool developed to simulate the real-time systems, networked control systems, communication models, and WSN [27]. The main feature of TrueTime is the possibility of co-simulation of the interaction between the real-world continuous dynamics and the computer architecture in the form of task execution and network communication. TrueTime computer block execute user-defined tasks and interrupt handlers representing, for instance, I/O tasks, control algorithms, and network drivers. The scheduling policy of the individual computer block is arbitrary and decided by the user. TrueTime allows simulation of context switching and task synchronization using events or monitors [27].

**Figure 5.**
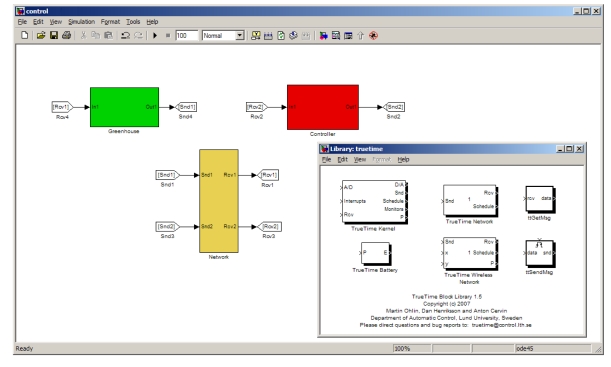
TrueTime window and implementation of event-based controller

Figure 5 shows the simulation scheme including the greenhouse model (in green), the wireless network (in yellow), and the controllers (in red). A real WSN based on IEEE 802.15.4 ZigBee protocol [28] has been simulated to perform the communications between wireless devices located at the greenhouse model and the simulated computer. Notice how in the simulation blocks are not connected in order to represent wireless communication.

### 4.2. Climate Monitoring and Data Transmission

Climate monitoring is vitally important to the operation in greenhouses and the quality of the collected information has a great influence on the precision and accuracy of control results. Currently, the agro-alimentary market field incorporates diverse data acquisition techniques. Normally, the type of acquisition system is chosen to be optimal for the control algorithm to be used. For traditional climate monitoring and control systems, all sensors are distributed through the greenhouse and connected to the device performing the control tasks. These equipments use time-based data sampling techniques as a consequence of using time-based controllers. Nowadays, commercial systems present more flexibility in the implementation of control algorithms and sampling techniques, especially WSN, where each node of the network can be programmed with a different sampling algorithm or local control algorithm with the main goal of optimizing the overall performance.

In modern control systems, it is common to use communication networks to transmit data between different control system blocks. Large amount of data are usually transmitted, the data required by the controller in each sampling time being especially critical. The most reasonable solution from an economical point of view is to make use of existing network structure, and to share the network resources between different services, for instance, using Ethernet networks. Sometimes, this solution can produce a big network traffic burden (in a typical greenhouse control system, all data are transmitted every minute or even faster) and introduce time delays in the delivery of the data packets. When the network loads increase, the probability of data losses increases too, and this factor can be very negative for control performance. In some extreme examples, the control system needs dedicated network structure to minimize the time delay and the data losses. On the other hand, the development of network structures in places with large distances, such as in greenhouse installations, can become very expensive and with a complicated management. Wireless networks present an economic and useful solution to this problem, and more concretely, WSN for recording data and control purposes.

However, most transceivers in WSN are battery powered and the power consumption is a critical parameter. Every transmission means bigger power consumption and thus these systems present the problem of limitation in the amount of data to transmit. A solution to this problem is the use of event-based sampling, that is, the level crossing sampling technique described in previous sections. This technique allows that only the necessary data will be transmitted and thus only the necessary power will be consumed.

As commented above, in this study the viability to implement a WSN based on IEEE 802.15.4 ZigBee protocol is analyzed and its combination with level crossing sampling in the control of greenhouse climatic. Simulation results were evaluated for a full crop campaign of 120 days. In this paper, eight days have been selected as representative to show the obtained results. The limits described in Table 1 were used for the level crossing sampling.

**Figure 6.**
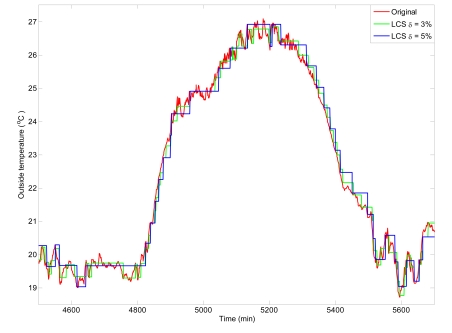
Effect of δ limit.

Table 2 presents the results obtained after simulation for the representative eight days, where the comparison of data transmission is presented for the greenhouse variables. The table compares the number of samples obtained and transmitted using level crossing sampling with a timed-based sampling where data is transmitted every minute (as usual in greenhouse control systems). As it can be observed, a considerable saving in transmission is obtained for both limits, *δ* = 3 % and *δ* = 5 %, obtaining transmission saving values over 90 % for most of the variables. Furthermore, it is observed that the amount of transmitted data is smaller when the *δ* limit is bigger. So, it is clear that the number of samples depends on 2 factors: the limit *δ* and the variable dynamics. The effects of the *δ* limit can be observed in Figure 6 where the transmission of the inside temperature signal is shown. As it can be noticed, the transmission data is smaller for *δ* = 5% but proving a bigger signal destruction.

**Figure 7.**
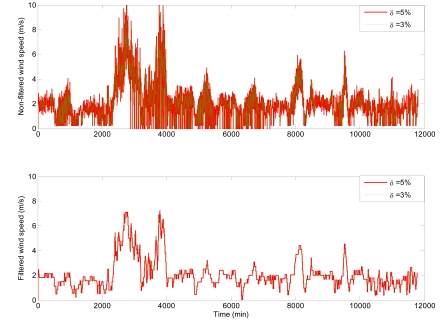
High-frequency dynamics.

On the other hand, the variable dynamics highly affects the number of samples taken. This can be observed for variables with high-frequency component such as the wind speed and direction. Figure 7 shows the transmission data for the wind speed. The transmission data from the sensors using level crossing sampling is shown on the top graphic, where a high transmission frequency is observed. However, in order to reduce the number of events created by this variable, the signal is filtered in the event generator before detecting and sending events to the controller. The bottom graphic shows how the number of samples-events has been considerable reduced after filtering the signal.

In conclusion, by choosing *δ* = 3%, it is possible to obtain a high reduction of acquired samples without relevant loss of information in the signals from a control design point of view. Therefore, this value of the *δ* limit will be used from now on.

### 4.3. Event-Based Control for Inside Temperature

**Figure 8.**
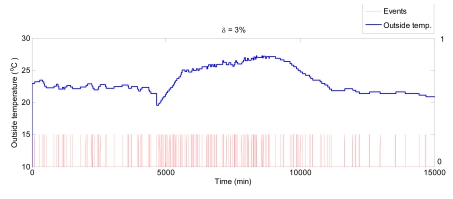
Event generation for outside temperature.

This section presents the simulation results obtained for the greenhouse climatic control problem. The control system works such as described in Figure 4, where the controller only calculates a control signal when an event happens. The event triggering is governed by an event generator which detects the possible events affecting the controller. For this simulation study, these events are represented by changes on: set-point, inside temperature, outside temperature, humidity, and wind speed. Figure 8 shows how the events are generated from changes in the outside temperature. Red lines indicate the event triggering by changes of the variable. The control results for the event-based control are presented as sampled signals in order to show better the influence of the events.

**Figure 9.**
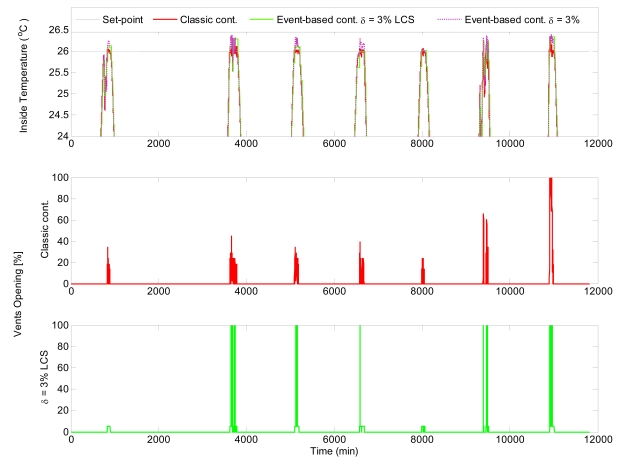
Event-based control with *δ* = 3 % versus time-based control during the diurnal period.

As discussed above, eight days are shown as representative of the simulation study. The temperature set-point was set at 26 °C and 17 °C for diurnal and nocturnal periods, respectively. Figures 9 presents the simulation results for diurnal period, with the purpose of showing up the influence of event based controller. This figure compares a time-based controller and an event-based controller with *δ* = 3 %.

**Figure 10.**
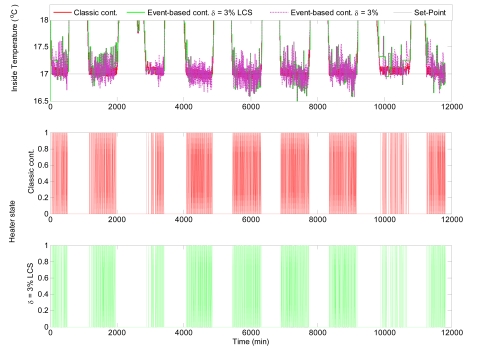
Event-based control with *δ* = 3 % versus time-based control during the nocturnal period.

**Figure 11.**
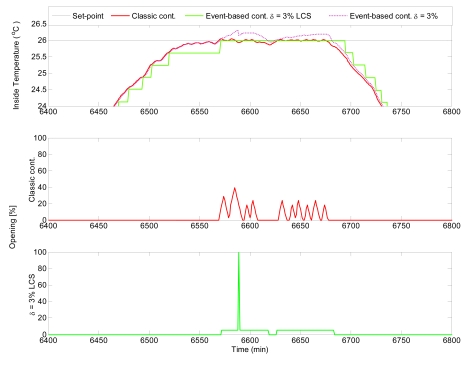
Control results for the fifth day using event-based control with *δ* = 3 %.

Figure 10 shows the comparison of the control results obtained using classic time-based control and event-based control for nocturnal period, where only nocturnal band of interest is shown. To present the influence of Level Crossing Sampling (LCS) in each figure, the inside temperature signal is shown after and before sampling. It can be seen that the time-based controller obtains better performance results but at expense of providing a major number of commutations of the control signal, such as observed in Figure 11, where the control results for the fifth day are shown. It can be observed that the inside temperature signal is within acceptable ranges for the event based controller (errors of less than 0.2 °C). The main advantage of event-based controller is reflected in the resulting control signals, where the number of commutation is considerably smaller than in the case of the classic controller.

**Figure 12.**
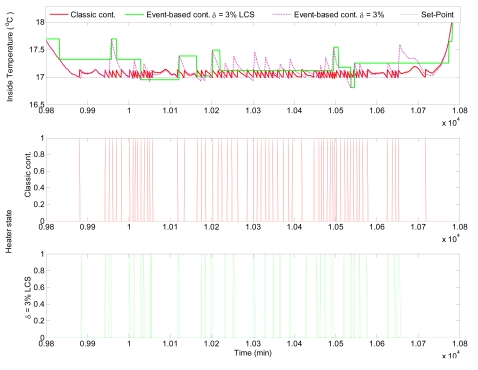
Control results for the eighth night using event-based control with *δ* = 3 %.

This fact can be better seen in Figure 12, which presents simulation results for the nocturnal period. A clear reduction in the control signal commutations is observed even using on/off controllers. However, in this case the control of the inside temperature using event-based control presents with bigger errors. This clearly established that a compromise between *δ* and the error value must be accomplished. In this sense, *δ* could be used as a tuning parameter for the compromise between control performance and reduction of the control signal changes.

From the previous results, it is observed that the number of changes in the control signals is much smaller for the event-based control cases. This fact is a key issue in the actuator lifetime, especially in greenhouses where the ventilation and heating systems are composed by mechanical actuators.

**Figure 13.**
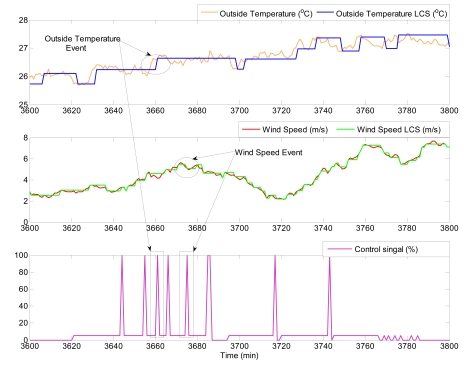
Event occurrence due to disturbances.

Another interesting result is to study the system response to load disturbances from an event-based point of view. Figure 13 shows two different situations where the event-based control system reacts against events produced by changes in the outside temperature and the wind speed. At *t* = 3,660 min, an event is generated due to a change in the outside temperature, resulting in a reaction of the controller to produce a new control signal. Notice that at this time, the wind speed is constant. On the other hand, at *t* = 3,673 min, the outside temperature is constant. However, the controller calculates a new control signal because an event comes as consequence of a change in the wind speed.

### 4.4. Event-Based Control for Inside Humidity

**Figure 14.**
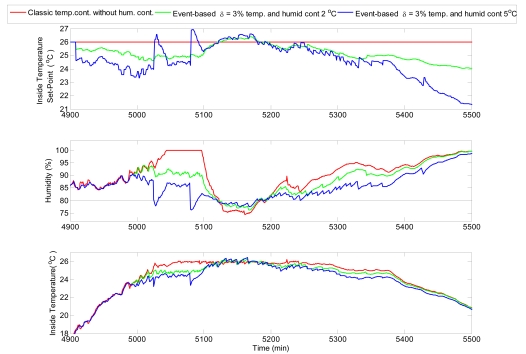
Comparison of simulation results for the humidity control.

Figure 14 demonstrates the simulation results for temperature and humidity control using event-based control with *δ* = 3 %. The control of humidity has been realized by the modification of the inside temperature set-point such as described in Section 2.3. In this case study, the aforementioned set-point was modified in the ranges ±2 and ±5 °C. The humidity reference was set to 80%, to be controlled with a tolerance of ±20%. For the first case, with a maximum modification of ±2 °C, it can be observed that the humidity control has a lower priority than the temperature controller. That is reflected in the humidity signal, which changed its value without reaching the set-point. The modification of the set-point allowed changing the humidity level around 10%, and it was controlled within the desired tolerance (±20%). In the second case, where the temperature set-point can be modified up to ±5 °C, the humidity controller is working with a higher priority than the temperature controller. Hence, it is observed that the inside humidity level is located near the set-point but it produces non-permissible higher errors in the control of the inside temperature.

### 4.5. Analysis from Numerical Results

**Figure 15.**
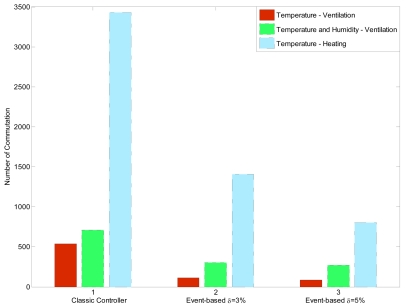
Number of changes produced by controllers for the inside temperature control.

After analyzing the results discussed above, a numerical comparative study was done based on the commutations or changes in the control signals and the control performance results. Figure 15 shows a comparison of the changes in the control signal for the time-based control, even-based control with *δ*=3 %, and event-based control with *δ*=5 %. As observed, for the diurnal temperature control with ventilation, the time-based control produced 539 changes in the control signal, while 113 and 86 changes where obtained for event-based control with *δ*=3 % and *δ*=5 %, respectively. For the nocturnal period, 3,429 changes was obtained for the classic controller, 1410 with *δ* = 3 %, and 804 with *δ* = 5 %. The activation of the humidity controller increases significantly the number of changes for the diurnal periods as a consequence of the changes in the inside temperature set-point. Thus, using event-based control it is possible to reduce the number of changes in more than the 80 % just for eight days. For a full crop campaign the reduction was around 82 %. This is a very important factor for the actuator lifetime and also from an economical point of view, since the use of electricity and fuel is considerably reduced.

**Figure 16.**
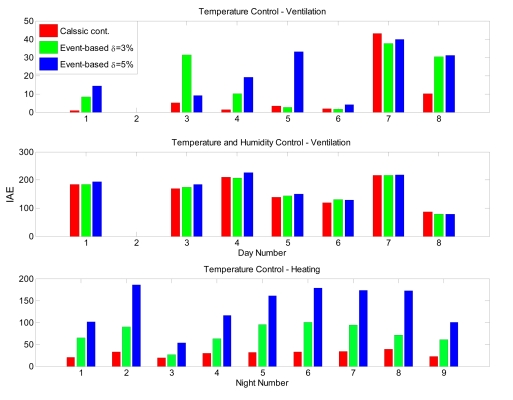
Performance comparison using IAE

On the other hand, as it was observed from the simulation results, the performance on the controlled variable is deteriorated when using event-based control. Hence, the integral absolute error (IAE) has been used as measurement to compare performance quality. Figure 16 shows the results of this comparison. As expected, the time-based controller presents better performance results, with the event-based controller giving *δ* = 3 % lower IAE values than the case with δ = 5 %. However, it can be seen that the performance results for *δ* = 3 % are not so far from those obtained using time-based control. Therefore, a compromise must be accomplished between performance and number of commutations. For the results presented in this paper, an event-based controller with *δ* = 3 % can be selected as a good choice since it presents acceptable performance results for the greenhouse climate control problem with a very important reduction in the control signals commutation.

Finally, an interesting result is that event-based controller with *δ* = 3 % provides better performance results than the time-based for some specific days, for instance, for the sixth day in the control with ventilation and for the eighth day in the control with humidity. This is because the time-based controller acts even for small error values, while the event-based one only works when the error is bigger than *δ*, thus keeping constant the error for longer periods of time.

## 5. Conclusions

This work presents an event-based control technique and its combination with WSN to solve the greenhouse climate control problem. Thanks to the combination of level crossing sampling with WSN, it was possible to obtain satisfactory results in the climate monitoring data transmission part. The event-based controller has allowed a considerable decrease in the number of changes in the control action and made possible a study of the compromise between quantity of transmission and control performance. The limit of the level crossing sampling has presented a great influence on the event-based control performance where, for the greenhouse climate control problem, a value of 3% has provided promising results.

On the other hand, event-based control reduced the number of changes by more than 80 % in comparison with a traditional time-based controller. This result is a key issue for greenhouses since it allows reduction of the electricity costs and increases the actuator lifetime. Further work is planned to test these ideas using other greenhouse control algorithms, e.g. fertirrigation control, where it would be also possible to obtain promising results from an economical point of view.

Furthermore, all simulated ideas will be implemented in a real greenhouse environment with the aim of verifying the obtained results in this simulation study. Notice that the proposed solutions can be easy implemented to any kind of WSN platform due to its small computational complexity.

## Figures and Tables

**Figure 1.**
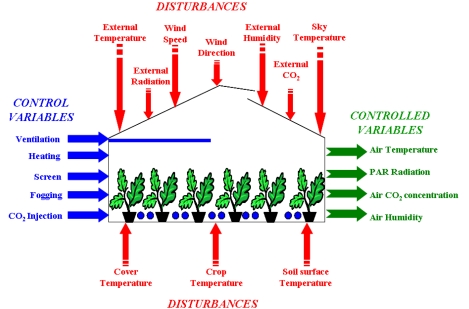
Climatic Control Variables.

**Table 1.** Limits for greenhouse variables.

**Variable**	**Limit (δ = 5%)**	**Limit (δ = 3%)**
Inside Temperature	0.60	0.36
Outside Temperature	0.61	0.36
Humidity	2	1.2
Solar Radiation	34.30	20.58
Wind Speed	0.53	0.31
Wind Direction	17.84	10.70

**Table 2.** Data transmission results.

**Variable**	**Time-based (samples)**	***δ* = 3% (samples)**	**Saving (%)**	***δ* = 5% (samples)**	**Saving (%)**
Inside Temperature	11808	469	96.02	279	97.63
Outside Temperature	11808	762	93.54	353	97.01
Humidity	11808	674	94.29	358	96.96
Solar Radiation	11808	826	93.00	553	95.31
Wind Speed	11808	5720	51.55	3715	68.53
Wind Direction	11808	5003	57.63	3255	72.43

## References

[1] van Straten G. (2007). What can systems and control theory do for agriculture?.

[2] Farkas I. (2005). Modelling and control in agricultural processes. Comput. Electron. Agric..

[3] Sigrimis N., Antsaklis P., Groumpos P. (2001). Special issue on Control advances in agriculture and the environment. IEEE Control Syst. Mag..

[4] King R., Sigrimis N. (2000). Computational intelligence in crop production. Comput. Electron. Agric..

[5] Sigrimis N., King R. (1999). Special Issue on Advances in greenhouse environment control. Comput. Electron. Agric..

[6] Narasimhan L.V., Arvind A., Bever K. (2007). Greenhouse asset management using wireless sensor-actor networks.

[7] Gonda L., Cugnasca C.E. (2006). A proposal of greenhouse control using wireless sensor networks.

[8] Zhu Y.W., Zhong X.X., Shi J.F. (2006). The design of wireless sensor network system based on Zigbee technology for greenhouses. J. Phys..

[9] Feng X., Yu-Chu T., Yanjun L., Youxian S. (2007). Wireless Sensor/Actuator Network Design for Mobile Control Applications. Sensors.

[10] Sandee J.H., Heemels W.P.M.H., van den Bosch P.P.J. (2005). Event-driven control as an opportunity in themultidisciplinary development of embedded controllers.

[11] Årzen K.J. (1999). A simple event-based PID controller.

[12] Åstrom K.J., Astolfi A., Marconi L. (2007). Analysis and Design of Nonlinear Control Systems. Event based control.

[13] Miskowicz M. (2003). The event-triggered sampling optimization criterion for distributed networked monitoring and control systems.

[14] Åström K.J., Wittenmark B. (1997). Computer controlled systems: Theory and design..

[15] Dormido S., Sánchez J., Kofman E. (2008). Sampling, event-based control and communication (in Spanish). Rev. Iber. Auto. Infor. Indu..

[16] Rodríguez F. (2002). Modeling and hierarchical control of greenhouse crop production (in Spanish). PhD thesis.

[17] Rodríguez F., Guzmán J.L., Berenguel M., Arahal M.R. (2008). Adaptive hierarchical control of greenhouse crop production. Int. J. Adap. Cont. Signal Process..

[18] Bot G.P.A. (1983). Greenhouse climate from physical processes to a dynamic model. PhD thesis.

[19] Kamp P.G.H., Timmerman G.J. (1996). Computerized environmental control in greenhouses. A step by step approach..

[20] Rodríguez F, Berenguel M., Arahal M.R. (2001). Feedforward controllers for greenhouse climate control based on physical models.

[21] Ellis P. (1959). Extension of phase plane analysis to quantized systems. IRE Trans. Automat. Control.

[22] Xia F., Zhao W. (2007). Flexible Time-Triggered Sampling in Smart Sensor-Based Wireless Control Systems. Sensors.

[23] Suh Y.S. (2007). Send-On-Delta Sensor Data Transmission With A Linear Predictor. Sensors.

[24] Miskowicz M. (2006). Send-On-Delta Concept: An Event-Based Data Reporting Strategy. Sensors.

[25] Miskowicz M. (2007). Asymptotic Effectiveness of the Event-Based Sampling According to the Integral Criterion. Sensors.

[26] Henriksson D., Cervin A., Årzen K.E. (2003). Truetime: Real-time control system simulation with matlab/simulink.

[27] Anderson M., Henriksson D., Cervin A., Årzen K.E. (2005). Simulation of Wireless Networked Control Systems.

[28] IEEE 802.15.4-2006 IEEE Standard for Information technology. Telecommunications and information exchange between systems. http://standards.ieee.org/getieee802/download/802.15.4-2006.pdf.

